# Evaluation of clinical variables according to follow-up times in COPD: results from ON-SINT cohort

**DOI:** 10.1080/20018525.2017.1394132

**Published:** 2017-11-02

**Authors:** José Luis López-Campos, Alberto Fernández-Villar, Cristina Represas Represas, Lucía Marín Barrera, Maribel Botana Rial, Cecilia López Ramírez, Ricard Casamor

**Affiliations:** ^a^ Unidad Médico-Quirúrgica de Enfermedades Respiratorias, Instituto de Biomedicina de Sevilla (IBiS), Hospital Universitario Virgen del Rocío/Universidad de Sevilla, Sevilla, Spain; ^b^ CIBER de Enfermedades Respiratorias (CIBERES), Instituto de Salud Carlos III, Madrid, Spain; ^c^ Servicio de Neumología, Complexo Hospitalario de Vigo, Sevilla, Spain; ^d^ Instituto de Investigación biomédica de Vigo (IBIV), Vigo, Spain; ^e^ Departamento Médico de Novartis Farmacéutica, Novartis España, Barcelona, Spain

**Keywords:** COPD, clinical presentation, progression, cohort, FEV_1_ decline

## Abstract

**Background**: COPD is a chronic disease traditionally associated with increased symptoms as lung function deteriorates. Follow-up times in previous cohort studies were limited to a few years. Interestingly, newer longer observational studies show a more comprehensive picture on disease progression over time. Therefore, the question on the relevancy of the follow-up time in cohort studies remains open.

**Methods**: The ON-SINT study is an observational, retrospective, nationwide, real-life cohort study, in which patients diagnosed with COPD were recruited between December 2011 and April 2013 by primary care (PC) and secondary care (SC) physicians. Patients were evaluated at the inclusion visit and at the initial visit when the diagnosis of COPD was first established. Distribution of lung function decline over the years was studied comparing those cases with longer follow-up times, with the median of the distribution as the cutoff point.

**Results**: The sample included 1214 patients of which 857 (70.6%) were recruited by PC and 357 (29.4%) by SC physicians. Median follow-up time was 6.26 years. Mean annual change in the complete cohort were –4.5 (222) ml year^–1^ for FVC and 5.5 (134) ml year^–1^ for FEV_1_. We confirm the variable distribution of FEV_1_ decline and found that longer follow-up periods reduce this variability. Of note, FEV_1_ decline was different between groups (shorter: 19.7 [180.4] vs longer: –9.7 [46.9]; *p *= 0.018). Further, our data revealed differences in the clinical presentation according to follow-up times, with special emphasis on dyspnea (OR: 1.035; 95%CI: 1.014–1.056), exacerbations (OR 1.172; 95%CI 1.045–1.315) and CAT scores (OR 1.047; 95%CI 1.019–1.075) being associated with longer follow-up times.

**Conclusions**: This study describes the impact of follow-up periods on lung function variability, and reveals differences in clinical presentation according to follow-up times, with special emphasis on dyspnea, exacerbations and CAT scores.

## Introduction

In 1977 Fletcher and Peto described the progressive loss of FEV_1_ in a working population,[1] suggesting the concept COPD as a progressive airflow obstruction.[2,3] Based on this and subsequent studies, the idea behind COPD is that of a progressive decrease in airflow due to chronic exposure to inhaled fumes and particles, which in turn was associated with an increase in symptoms as FEV_1_ declined.[4] However, more recent research is challenging this concept, since FEV_1_ does not always decline, and this decline may vary,[5] as various rates of FEV_1_ decline have been described depending on the severity of the disease,[6] and symptoms do not correlate with FEV_1_ at the individual level.[7,8]

Should the idea of an association between decline in lung function and worsening of symptoms be correct, then patients with longer follow-ups would have a more severe disease status, poorer lung function and more impaired health status. However, evidence on the clinical presentation of the disease over time may not support this statement. Follow-up times in initial cohort studies were restricted to a few years,[5,7] hence longer observational periods were required to yield more solid conclusions. More recently, longer studies have provided new information on lung function decline, evaluating its progression and additionally setting the importance of lung development at earlier ages as a determinant factor of lung function impairment in adult life.[9] Further, individualized prediction equations have been proposed for lung function decline at individual level,[10] which may in turn be associated with specific gene susceptibility together with tobacco smoke.[11] The results of these studies with longer follow-up periods give a more comprehensive picture of COPD progression over time. Interestingly, the specific role of follow-up time on the evaluation of the progression of the disease becomes of interest.

The ON-SINT study (clinical presentation, diagnosis, and outcome of chronic obstructive pulmonary disease) is an observational, retrospective, cohort study that aims to evaluate the clinical and functional presentation of COPD at diagnosis, as well as its progress over time, both in primary care and specialized care. Based on an analysis of the ON-SINT cohort database, the present study aims to evaluate differences between cases with shorter and longer follow-ups in terms of lung function decline and clinical presentation. The results of this study will help understand the temporal changes in the COPD clinical setting.

## Methods

The methodology of the ON-SINT study has been extensively described.[12] Briefly, it is an observational, nationwide, real-life, retrospective, cohort study, in which patients diagnosed with COPD were recruited between December 2011 and April 2013 by primary care (PC) and secondary care (SC) physicians. Consecutive patients aged >40 years who were smokers or ex-smokers with a history of >10 pack-years, diagnosed with COPD, with a complete clinical history of respiratory symptoms, able to complete the CAT questionnaire, and who gave their written informed consent were selected to participate in the study. Ethical approval was granted by the Institutional Review Board from *Servicio Gallego de Salud* (SERGAS), registry number 2011/359. In order to record real-life clinical behavior of participant doctors, the only exclusion criterion considered in the study protocol was participation in any other clinical trial at the time of inclusion. In addition, in order to make a real-life evaluation, patients were recruited by PC and SC physicians with no matching for gender, age, lung function or any clinical features. Sample size was calculated according to the prevalence and the degree of under-diagnosis of COPD in Spain.[13] A total of 1440 patients with COPD was calculated to constitute a 0.1% sample of the study population, assuming a 10% loss of patients with no valid information. Although uniform distribution within the country was planned, including all regions, the selection of participant investigators was voluntary, with no intention to achieve representative sampling.

During the inclusion visit, patients underwent a clinical evaluation including screening for COPD risk factors. Smoking history was collected, including current status and cumulative consumption expressed in pack-years. Exposure to other substances was categorized into three groups: occupational dust and chemicals, biomass fuels, or other exposures. The CAT questionnaire was administered to all participants at the inclusion visit. The questionnaire was self-administered or administered by the investigator in case of any reading, writing or sight difficulties. If more than two questions were unanswered, the questionnaire was considered invalid.

After the inclusion visit, the medical record was reviewed to identify the diagnostic visit as that in which COPD was first diagnosed. Clinical information on this diagnostic visit, as well as spirometric values, were extracted and considered as baseline values.

### Statistical computations

Although subjects were recruited by respiratory and primary care physicians, all patients recruited by respiratory physicians were also followed up by their general practitioners, so the comparison between PC and SC was impractical and would draw confounding results. Consequently, all patients were analyzed together. The statistical analysis was performed with IBM SPSS Statistics (IBM Corporation, Somers, NY, USA), version 24.0. Absolute and relative frequencies for categorical questions were used to describe the variables. Quantitative data are expressed as mean (standard deviation). To evaluate the differences over time, we divided the sample according to time from diagnosis either above or below the median. Cross-sectional differences between patients above or below the median were calculated using the chi-squared test or the Student *t*-test for unpaired values, previously evaluating the similitude of the variance with the Levene’s test. Variables with *p*-values below 0.1 were entered in a stepwise forward multivariate binomial logistic regression analysis with ‘follow-up longer than the median’ as the dependent variable, and expressing the results as odds ratio (OR) and 95% confidence intervals (95%CI). Longitudinal changes in spirometric values were assessed by the paired *t*-test. FVC and FEV_1_ changes were calculated dividing the difference between by the number of follow-up years for every case, and this was represented graphically in a bar plot. Longitudinal changes in therapeutic groups were evaluated by the McNemar test. The alpha error was set at 0.05.

## Results

**Figure 1.**
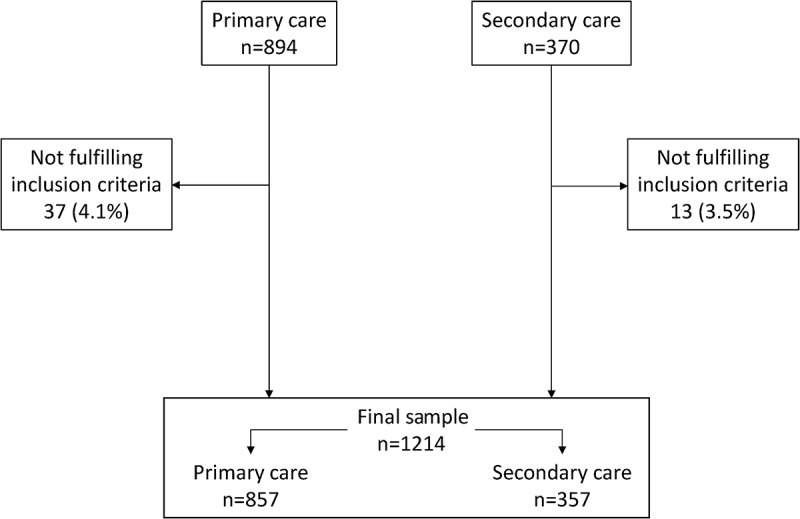
Flowchart of patients included in study.

The sample included 1214 patients of which 857 (70.6%) were recruited by 263 PC physicians and 357 (29.4%) by 93 SC physicians (Figure 1). The characteristics of the cases included are summarized in Table 1. Tobacco, occupational and biomass exposure have been previously reported.[14] Briefly, 1012 (83.4%) had tobacco as the only risk factor and 202 (16.6%) had additional ones, mainly 174 (14.3%) with occupational gases and 32 (2.6%) with biomass exposure. Median follow-up time was 6.26 years. The crude differences between cases with a prolonged follow-up time (beyond the median) and those with shorter follow-up times are summarized in Table 1.

**Table 1.** Characteristics of patients included in ON-SINT cohort at inclusion visit.

Variable	Total sample *n *= 1214	Follow-up < median *N *= 607	Follow-up > median *N *= 607	*P*-value
Male (*n*)	955 (78.7)	460 (75.8)	495 (81.5)	0.011
Age (years)	66.4 (9.7)	63.8 (9.5)	69.0 (9.2)	< 0.001
Body mass index (kg m^–^^2^)	27.7 (4.1)	27.5 (3.9)	28.0 (4.3)	0.050
Active smoker (*n*)	318 (26.2)	189 (31.1)	129 (21.3)	< 0.001
Tobacco history (pack-years)	36.3 (20.8)	35.8 (20.3)	36.8 (21.2)	0.443
Other substances besides tobacco (*n*)	202 (16.6)	87 (14.3)	115 (18.9)	0.037
Comorbidities (Charlson Index)	1.6 (1.5)	1.3 (1.3)	1.9 (1.6)	< 0.001
Sleep apnea (*n*)	258 (21.3)	104 (17.1)	154 (25.4)	< 0.001
Dyslipidemia	603 (49.7)	280 (46.1)	323 (53.2)	< 0.001
Arterial hypertension (*n*)	757 (62.4)	336 (55.4)	421 (69.4)	< 0.001
Dyspnea (modified MRC scale)	1.5 (0.8)	1.4 (0.8)	1.6 (0.8)	0.001
Exacerbations in previous year (*n*)	2.3 (1.9)	1.9 (1.7)	2.7 (2.1)	< 0.001
FVC post-bronchodilation (%)	74.5 (19.5)	77.3 (18.8)	71.6 (19.7)	< 0.001
FEV_1_ post-bronchodilation (%)	61.6 (20.2)	63.7 (19.6)	59.6 (20.6)	0.006
CAT score (points)	18.3 (7.6)	16.3 (7.5)	20.2 (7.1)	< 0.001
Performed any rehabilitation program (*n*)	181 (14.9)	68 (11.2)	113 (18.6)	0.001
Long-term oxygen therapy (*n*)	181 (14.9)	53 (8.7)	128 (21.1)	< 0.001
Surgical interventions for COPD (*n*)	37 (3.0)	13 (2.1)	24 (4.0)	0.162

**Figure 2.**
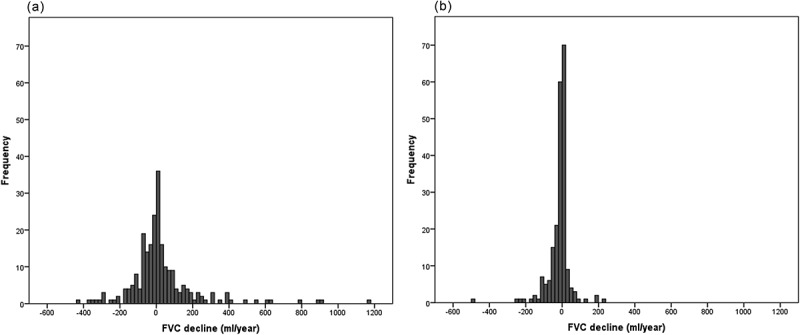
Post-bronchodilator FVC decline in 20 ml year^–1^ intervals for: (a) cases with follow-up times shorter than the median, and (b) cases with follow-up times longer than the median.

**Figure 3.**
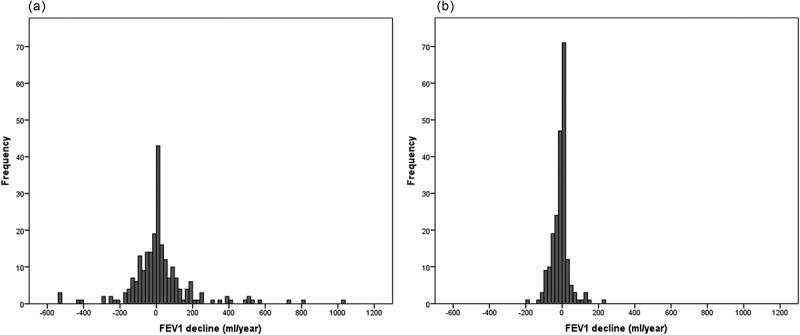
Post-bronchodilator FEV_1_ decline in 20 ml year^–1^ intervals for: (a) cases with follow-up times shorter than the median, and (b) cases with follow-up times longer than the median.

Lung function progression parameters for both follow-up groups are summarized in Table 2. Cases with longer follow-up times presented a significant change from baseline, whereas those with follow-up times below 6.26 years did not. Mean annual changes in the complete cohort were –4.5 (222) ml year^–1^ for FVC and 5.5 (134) ml year^–1^ for FEV_1_. Differences in FVC decline between those with a follow-up time shorter than the median (5.7 [301.7]) and those longer (–15.9 [62.3]) did not reach the statistical significance (*p *= 0.282). Of note, FEV_1_ decline was different between groups (shorter: 19.7 [180.4] vs. longer: –9.7 [46.9]; *p *= 0.018). However, FVC and FEV_1_ changes varied considerably over the follow-up period (Figures 2 and 3). Interestingly, the dispersion of the data was considerably narrower with longer follow-up times.

**Table 2.** Changes in lung function parameters over time.

	At diagnostic visit	At inclusion visit	*P*-value *
Follow-up time < median			
FVC (ml)	2931 (1081)	2949 (1140)	0.611
FVC (%)	76.7 (18.0)	77.6 (18.6)	0.219
FEV_1_ (ml)	1883 (717)	1916 (800)	0.177
FEV_1_ (%)	62.9 (18.1)	64.0 (18.9)	0.151
FEV_1_/FVC	62.7 (14.3)	63.3 (15.7)	0.340
Follow-up time > median			
FVC (ml)	2791 (1160)	2510 (1043)	< 0.001
FVC (%)	74.2 (18.6)	72.0 (18.2)	0.008
FEV_1_ (ml)	1879 (820)	1763 (827)	< 0.001
FEV_1_ (%)	60.1 (19.4)	59.1 (20.8)	0.023
FEV_1_/FVC	65.4 (15.7)	64.6 (16.9)	0.265

* Calculated by paired *t*-test

Changes in pharmacological treatments during the follow-up are summarized in Table 3. Altogether there was an increase in the percentage of patients receiving double bronchodilator therapy and triple therapy.

**Table 3.** Changes in pharmacological treatments during the follow-up.

	At diagnostic visit	At inclusion visit	*P*-value *
No long-acting inhaled medication	578 (47.6)	130 (10.7)	< 0.001
One LABD	173 (14.3)	188 (15.5)	0.344
ICS alone	91 (7.5)	45 (3.7)	< 0.001
LAMA+LABA	66 (5.4)	261 (21.5)	< 0.001
ICS + 1 LABD	162 (13.3)	185 (15.2)	0.158
Triple therapy	144 (11.9)	405 (33.4)	< 0.001
Methylxanthines	79 (6.5)	69 (5.7)	0.399
Roflumilast	10 (0.8)	124 (10.2)	< 0.001
Mucolytics	245 (20.2)	331 (27.3)	< 0.001

* Calculated by the McNemar test

The results of the multivariate analysis are shown in Table 4. After adjusting for all confounders, only age, number of exacerbations in previous year and CAT scores were associated with a prolonged follow-up beyond 6.26 years.

**Table 4.** Multivariate analysis of factors associated with longer follow-up times.

	Crude		Adjusted	
	OR	95%CI	OR	95%CI
Male (*n*)	1.441	1.091–1.905		
Age (years)	1.054	1.039–1.070	1.035	1.014–1.056
Body mass index (kg m^–^^2^)	1.028	1.000–1.056		
Active smoker (*n*)	0.596	0.459–0.773		
Other substances besides tobacco (*n*)	1.397	1.030–1.894		
Comorbidities (Charlson Index)	1.398	1.270–1.539		
Sleep apnea (*n*)	1.676	1.267–2.216		
Dyslipidemia	1.389	1.106–1.743		
Arterial hypertension (*n*)	1.960	1.543–2.489		
Dyspnea (modified MRC scale)	1.253	1.091–1.440		
Exacerbations in previous year (*n*)	1.207	1.120–1.301	1.172	1.045–1.315
FVC post-bronchodilation (%)	0.985	0.977–0.993		
FEV_1_ post-bronchodilation (%)	0.990	0.983–0.997		
CAT score (points)	1.065	1.046–1.085	1.047	1.019–1.075
Performed any rehabilitation program (n)	1.853	1.337–2.567		
Long-term oxygen therapy (n)	2.809	1.992–3.960		

## Discussion

The present study analyzes the changes over time of different clinical outcomes with special emphasis on lung function decline. The main findings are: (1) we confirm the fluctuating distribution of FEV_1_ decline, with rapid and slow decliners, and report cases with longer follow-up periods where this variability decreases; (2) we found differences in the clinical presentation according to follow-up times with special emphasis on dyspnea, exacerbations and CAT scores; and (3) we describe the limited mean change in lung function from baseline.

Cohort studies are a powerful tool to evaluate the progression of chronic diseases like COPD. In our case, certain aspects must be borne in mind if we are to interpret our results correctly. Although representative sampling was not being sought, the main strength is the national coverage of the sample with PC and SC recruiting patients using a standardized questionnaire for all centers. Of note, ON-SINT is a retrospective cohort study and cases with longer follow-up times might represent an additional challenge to record all the clinical information at the diagnostic visit. Researchers were alerted to this potential limitation and were encouraged to complete the information as accurately as possible by choosing the visit in which the diagnosis was established. Finally, due to the methodology of the study, information was recorded at two timepoints. Therefore, we did not record information of what happened in between, e.g. tobacco changes, that may explain the different progression of lung function.

Description of FEV_1_ decline has been reported in several previous studies. The *Evaluation of COPD Longitudinally to Identify Predictive Surrogate End-points* (ECLIPSE) study evaluated 2163 patients over three years. These authors found that the mean rate of change in FEV_1_ was a decline of 33 ml per year, with significant variation in the levels of change. Interestingly, the rate of change was not associated with the number of FEV_1_ measurements.[15] The BODE cohort evaluated 1198 stable, well-characterized patients with COPD, monitored from 1997 to 2009. These authors also found considerable variability in FEV_1_ decline, similar to ours.[5,16] More recently, several studies have evaluated the progression of FEV1, confirming previous findings [17] and adding a potential effect on lung growth as a marker of the final lung functioning in adult life.[9] In particular, Lange et al described the progression of FEV1 in three independent cohorts, defining four trajectories according to FEV_1_ at the beginning and the end of the cohort. Interestingly, although the long-term progression was different for each trajectory, the distribution of the observed declines in FEV_1_ in the four trajectory categories resemble ours and showed substantial variability and overlap between trajectories.[9] More recent studies have confirmed this different trajectories [17] as they were already described in the past.[18] Our study corroborates this variability in the progression of FEV_1_ decline, by showing patients with lung function that worsens and improves over time. Additionally, by comparing follow-up times we here provide additional view of the progression of the disease in regards with lung changes over time not previously reported by these observational studies. One new and interesting finding was the establishing of an inverse relationship between time tracking and variability of FEV_1_ decline. This is interesting because some previous analysis lasted only a few years.[5,7,19] According to our data, differences are found between our study groups with a cutoff of 6.2 years, which suggests that cohorts should have longer follow-ups if we wish to adequately establish the variability in FEV_1_ decline.[20]

Changes in pharmacological prescription over time show an increase in more intense therapeutic combinations in favor of double and triple therapies. This increase is in line with previous publications on inhaled therapies use.[21] Our results evaluated these combinations irrespective of whether they were administered in one or two inhalers, since it has been shown to have a similar efficacy.[22,23] Roflumilast increased since it was recently commercialized in our country and only cases with shorter follow-up times had the possibility to receive it at baseline.

How the disease presents depending on follow-up times reveals some differences for patients with longer follow-up times. Although the initial bivariate analysis showed differences between longer and shorter follow-up times, after adjusting for confounders, only dyspnea, number of exacerbations and CAT scores remained as significant in the model. This confirms previous findings on the importance of these three dimensions of the disease.[24] Chronic and progressive dyspnea is the most characteristic symptom of COPD with a major impact on health status [25] and disease prognosis.[26] The clinical impact of exacerbations on various aspects of the disease has been demonstrated in numerous studies.[27] We know that exacerbations produce worsening symptoms, increase morbidity,[28,29] impair quality of life,[30] increase functional impairment,[31] relate to various comorbidities,[32,33] worsen clinical findings reported by patients,[34] and, ultimately, relate to the progression of disease [35] with a major impact on prognosis.[36] In addition, exacerbations have a significant impact on the financial burden of the disease for the health system.[37] Therefore, reducing exacerbations has been established as a priority in the treatment of COPD.[38] Finally, CAT is a recently developed questionnaire to quantify the impact of COPD on a patient’s life, and how this changes over time.[39] Despite its short life, this instrument has been validated in several different scenarios including stable disease,[40] during exacerbations,[41] associated with certain comorbidities,[42] and after some interventions such as pulmonary rehabilitation.[43] Thus, the CAT has become one of the main instruments in the evaluation of health status in COPD. Consequently, it has been incorporated into the GOLD strategy.[24]

It is interesting to note that some of the variables considered initially did not remain in the multivariate model, including gender, active smoking and comorbidities. Despite increased diagnosis of COPD in women and the different disease expression,[44] our data failed to find an association with follow-up time. This is probably because the majority of our patients were males and peak COPD prevalence rates in women have yet to be seen. Active smoking is decreasing in developed countries and the prevalence of tobacco smoking is similar to that described for our country. However, in Spain smoking prevalence has been decreasing slightly but with no significant change in the slope over the past decades.[45] Comorbidities are generally expected to increase with time. However, our cohort did not reveal any association between follow-up times and the Charlson Index or other comorbidities including sleep apnea, dyslipidemia, or systemic arterial hypertension. This probably reflects age as a confounding factor, which was retained in the model. Additionally, other factors like treatments or smoking habit were not associated with lung function decline in our cohort. The impact of different treatments and specifically long-acting bronchodilators on FEV_1_ decline has not been consistently demonstrated in previous high-powered clinical trials.[46] Although the relationship of tobacco with lung function decline has been well established,[47] we did not find this association. We are now starting to understand the genetic determinants that may drive this different response after quitting tobacco.[11]

## Conclusions

In summary, this study evaluates changes in different clinical outcomes and lung function over time. It confirms the variable distribution of FEV_1_ decline and describes the importance of follow-up periods for assessing this variability, with differences in clinical presentation according to follow-up time, with special emphasis on dyspnea, exacerbations and CAT scores. The findings of our study are relevant for investigators interested in exploring the follow-up times needed for cohort studies of a chronic, slowly progressing and debilitating clinical condition like COPD.
